# Supplementary material for: OsABF1 Represses Gibberellin Biosynthesis to Regulate Plant Height and Seed Germination in Rice (Oryza sativa L.)
Source: Int J Mol Sci. 2021 Nov 12;22(22):12220. doi: 10.3390/ijms222212220 (PMC8622533; doi:10.3390/ijms222212220)
Supplement: Supplementary file 1 [file ijms-22-12220-s001.zip › ijms-1320158-supplementary.pdf]

Supplement figures

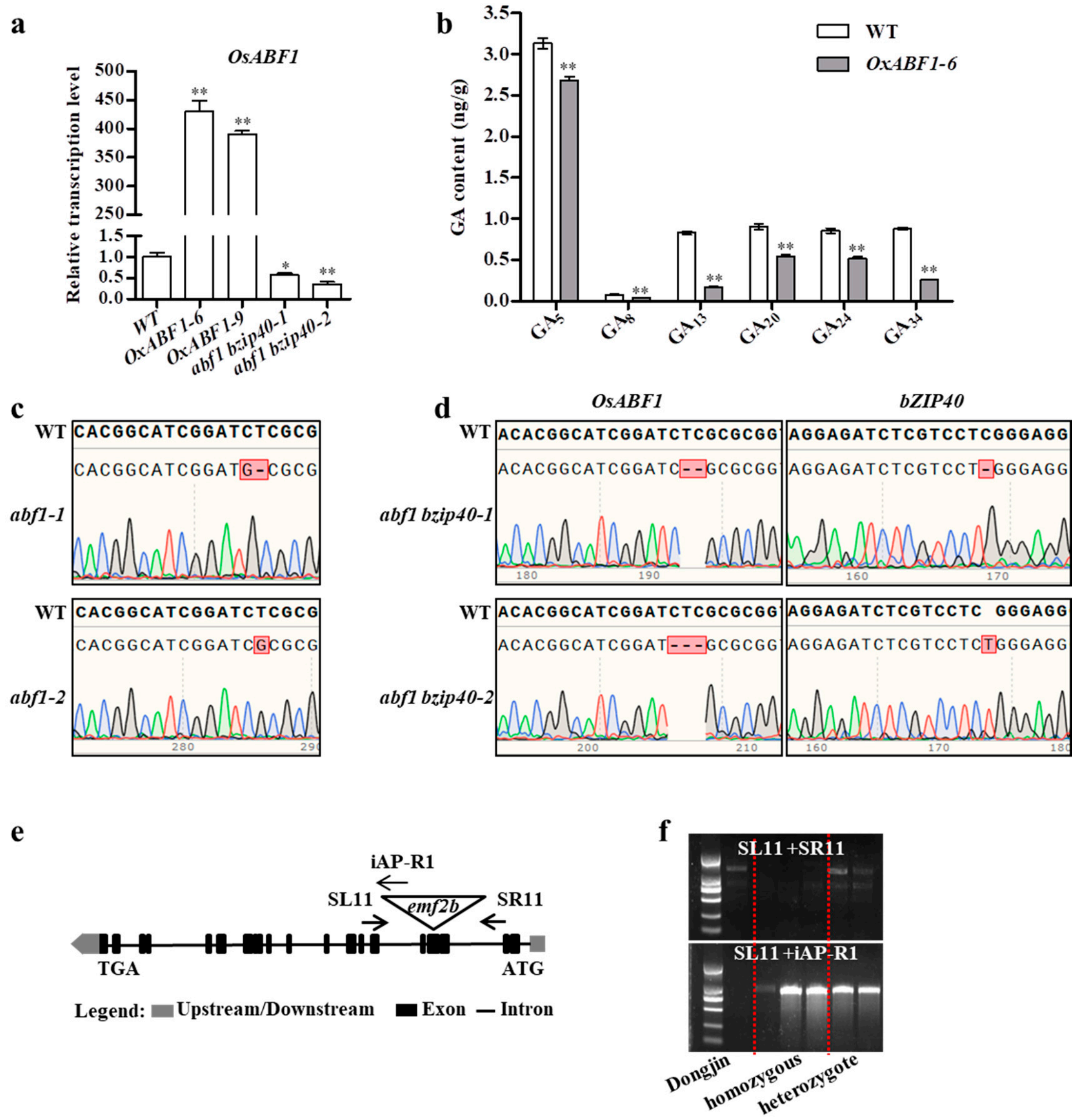

Figure S1. Genotypes of mutant and over-expression lines. (**a**) qRT-PCR analysis of *OsABF1* transcription in WT, *OxABF1-6*, *OxABF1-9*, *abf1bzip40-1* and *abf1bzip40-2* leaves at tillering stage. (**b**). Quantification of GAs derivative of WT and *OxABF1-6* seedlings analyzed with liquid chromatography-tandem mass spectrometry. Error bars represent means ± SD (n = 3). *, $P < 0.05$, **, $P < 0.01$ by Student's *t*-test analysis. (**c**) Sanger sequencing of the target sites of single mutant lines: *abf1-1* and *abf1-2*. (**d**) Sanger sequencing of the target sites of double mutants: *abf1bzip40-1* and *abf1bzip40-2*. (**e**) Gene structure of *OsEMF2B*. (**f**) T-DNA detection by gel electrophoresis, primers SL11 and SR11were used in up gel; primers SL11 and iAP-R1 were used in bottom gel.

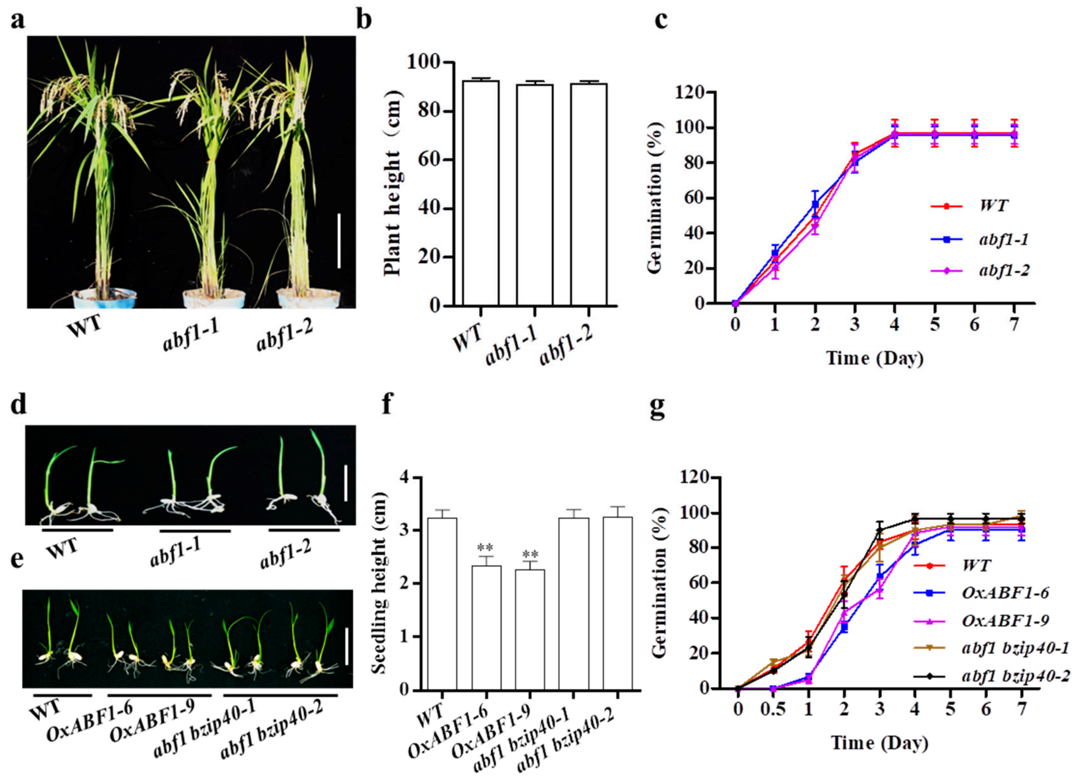

Figure S2. Phenotypes of *abf1*, *abf1bzip40* and *OsABF1* over-expression lines. (**a**) Phenotypes of *abf1-1* and *abf1-2*. (**b**) Plant height of *abf1-1* and *abf1-2*. (**c**) Seed germination time courses of *abf1-1* and *abf1-2*. (**d**) After 7 days' seed germination phenotypes of WT, *abf1-1* and *abf1-2*. (**e-g**), seed germination phenotypes (**e**), seedlings height (**f**) and Seed germination time courses of WT, *OxABF1-6*, *OxABF1-9*, *abf1 bzip40-1 and abf1 bzip40-2* (**g**). **b**-**c** and **f**-**g**, error bars represented means ± SD (n = 20). **, P< 0.01 by Student's t-test analysis.

Table S1. Sequences of primers used in this study.

Name	Gene ID	Primer sequence (5' to 3')	Usage
OxABF1-F	LOC_Os01g64730	TTACTTCTGCACTAGGTACCATGATGGCGTCGAGGGTGAT	over-expression
OxABF1-R		GAATTCCCGGGGATCCCTACCACTCCATCGAGTTTG	
OsABF1-U3-F		ggcACACGGCATCGGATCTCGCG	Crispr-cas9 mutation
OsABF1-U3-R		AAACCGCGAGATCCGATGCCGTG	
CrOsABF1-test F		TCCGTAATAAGACCACCAACCT	mutation site detection
CrOsABF1-test R		CGGCATGTCGGCGTAGAT	
bzip40-U6a-F	LOC_Os05g36160	gccGAGGAGATCTCGTCCTCGGG	Crispr-cas9 mutation
bzip40-U6a-R		AAACCCCGAGGACGAGATCTCCT	
Crbzip40-test F		AGGAGCACCAGCAGCGGCATCG	mutation site detection
Crbzip40-test R		CGTCATCTCAGGCCCGCCACCC	
Ubi-qRT-F	GenBank accession No.AF184280	GCTCCGTGGCGGTATCAT	qRT-PCR
Ubi-qRT-R		CGGCAGTTGACAGCCCTAG	
OsABF1-qRT-F	LOC_Os01g64730	CTGATGGATCCGATGGACCG	
OsABF1-qRT-R		GCGACTCGAGCTCAGCAATA	
OsABF1-F	LOC_Os01g64730	CTGGTTCCGCGTGGATCCATGATGGCGTCGAGGGTGAT	Pull down
OsABF1-R		CTCGAGTCGACCCGGGAATTCCTACCACTCCATCGAGTTTG	
OsEMF2b-F	LOC_Os09g13630	TGGGT CGCGG ATCCG AATTCATGTGCCGCCACCAGCCA	
OsEMF2b-R		TCGAGTGCGGCCGCAAGCTTTCAATTTTTCTTTGGATCCG	
OsABF1-F	LOC_Os01g64730	ATTATGCCTCTCCCGAATTCATGATGGCGTCGAGGGTGATGG	Y1H
OsABF1-R		GAAGTCCAAAGCTTCTCGAGCTACCACTCCATCGAGTTTGTT	
SD1- F	LOC_Os01g66100	CTTGAATTCGAGCTCGGTACCTTGCAATAACCGCGTGGTTT	
SD1-R		ATACAGAGCACATGCCTCGAGGAGATTGGAGATGAGAAGTG	
OsABF1-F	LOC_Os01g64730	TGGCCATGGAGGCCGAATTCATGATGGCGTCGAGGGTGAT	Y2H
OsABF1-R		CGCTGCAGGTCGACGGATCCCTACCACTCCATCGAGTTTG	
OsEMF2b-F	LOC_Os09g13630	CCATG GAG GCC ATG GAA TTCATGTGCCGCCACCAGCCA	
OsEMF2b-R		AGCTCGAGCTCGATGGATCCAATTTTTCTTTGGATCCG	
OsABF1-F	LOC_Os01g64730	GTAGTCACGTGACGTCCGGAATGATGGCGTCGAGGGTGA	BIFC
OsABF1-R		GCTCCACCAGAACCTCCGGACCACTCCATCGAGTTTGT	
OsEMF2b-F	LOC_Os09g13630	GTGGAGGTGGGTCAGGATCCATGTGCCGCCACCAGCCA	
OsEMF2b-R		AGGGCGCGCCCCATGGATCC ATTTTTCTTTGGATCCGAGC	
OsABF1-F	LOC_Os01g64730	TCTCTAGAACTAGTGGATCC ATGATGGCGTCGAGGGTGA	Luciferase activity
OsABF1-R		ATAAGCTTGATATCGAATTC CTACCACTCCATCGAGTTTG	
SD1-F	LOC_Os01g66100	CCCAAGCTTTTGCAATAACCGCGTGGTTT	

SD1-R		GAAGATCTTCGAGATTGGAGATGAGAAGTG	
SD1 *Pro*-1-F	LOC_Os01g66100	AGCTAGCTTGATCGTGGCTC	Chip -PCR
SD1 *Pro*-1-R		ACCACCCATGCACCATACAG	
SD1 *Pro*-2-F		ATCTGTCTCGTAACAGCCCC	
SD1 *Pro*-2-R		AACCTCTGGTAGCTTATCGATTT	
SD1 *Pro*-3-F		CCGGTGTTACACGTGCTCTA	
SD1 *Pro*-3-R		CACGTGGAAAACTAAACCTCTGG	
SD1 *Pro*-4-F		ACCAGAGGTTTAGTTTTCCACG	
SD1 *Pro*-4-R		GCGATCGCCTAAACTAGCCT	
SD1 *Pro*-5-F		ATGTCTGTCCAGTGGCAACC	
SD1 *Pro*-5-R		GCAGGAGGGTGGGGTATTTG	
SD1 *Pro*-6-F		GTGCAACTACTACCCGCCAT	
SD1 *Pro*-6-R		AAGGTGTCGCCGATGTTGAT	
Actin-F	LOC_Os03g50885	GAAATGGAGACTGCCAAGACC	
Actin-R		TTGGCAATCCACATCTGCTG	
